# Restorative Rehabilitation of a Patient with Dental Erosion

**DOI:** 10.1155/2017/9517486

**Published:** 2017-07-30

**Authors:** Mohammed Thamer AlShahrani, Satheesh B. Haralur, Mohammed Alqarni

**Affiliations:** ^1^Saudi Board of Restorative Dentistry, College of Dentistry, King Khalid University, Abha, Saudi Arabia; ^2^Department of Prosthodontics, College of Dentistry, King Khalid University, Abha, Saudi Arabia; ^3^College of Dentistry, King Khalid University, Abha, Saudi Arabia

## Abstract

Dental erosion is the chemical dissolution of the tooth structure. Factors like eating disorders and gastrointestinal diseases are recognized as intrinsic factors for dental erosion. Advanced stages of dental erosion extensively damage the tooth morphology, consequently affecting both esthetics and functions. Reports indicate the growing prevalence of erosion, and hence knowledge of restorative rehabilitation of tooth erosion is an integral part of the contemporary dental practice. This clinical report describes an adult patient with gastroesophageal reflux induced dental erosion involving the palatal surface of the maxillary anterior teeth. The extensive involvement of the palatal surfaces compromised the esthetics, incisal guidance, and functional occlusal efficiency. Indirect all-ceramic restorations were utilized to restore the esthetics and occlusal reconstruction. In conclusion, patients affected by severe dental erosion require prosthetic rehabilitation besides the management of the associated medical condition.

## 1. Introduction

Dental erosion is defined as the loss of tooth tissue through dissolution by acid without the involvement of bacteria. It is among the most common dental diseases along with periodontal diseases and dental caries [1]. The prevalence of dental erosion varies according to geographical areas, countries, and ages [2, 3]. According to previous surveys, preschool children in the age group of 2–5 years showed erosion in 6–50% of the cases [4, 5]. The sign of erosion in an adolescent group at the age between 9 and 17 years was at 11–100% [6], while the adults group showed prevalence between 4 and 82% [7].

The alarming fact is the increase in prevalence of dental erosion across all age groups [8]. The increased incidence is attributed to the changes in the diet and social and oral hygiene habits. As a large section of the society is affected, there is an obligation on restorative dentists to be well aware about the etiological factors, diagnosis, management, and prevention of dental erosion.

Etiological factors are mainly divided into intrinsic and extrinsic factors. The extrinsic factors are increased consumption of acidic beverages [9], food and candies [10], chewing gums, and medications. Industrial workers exposed to acidic fume or aerosols, professional wine tasters, and competitive swimmers are also at risk of dental erosion [11]. The principal intrinsic factor is the presence of gastric juice in the mouth due to frequent vomiting or regurgitation disorders [12]. Vomiting disorders include eating disorders [13] like bulimia and anorexia nervosa and medical conditions of the gastrointestinal, metabolic [14], neurological, and central nervous system disorders. Pregnancy-induced vomiting [15] and psychogenic vomiting syndrome are also reported to cause dental erosion.

Gastroesophageal reflux (GERD) is a common condition affecting a large percentage of the population. It is recognized as an involuntary retrograde movement of gastric contents into the mouth due to relaxation of the upper esophageal sphincter. Hydrochloric acid produced by parietal cells of the stomach is the major cause of intrinsic dental erosion. The pH and titratability of gastric acid are significantly higher than those of dietary acid, leading to extensive damage to the tooth structure [16]. According to the dental literature, 5–47.5% of GERD patients are affected by dental erosion [17, 18]. Dental erosion with intrinsic factors predominantly affects the palatal surfaces of maxillary anterior teeth with intact cervical enamel rim [19]. The progression of erosion involves cupping of posterior teeth and loss of incisal edges of anterior teeth. The patient affected by severe erosion requires extensive restorative rehabilitation.

Restoration of the worn dentition needs an analysis of the degree of structural damage. Restorative dentists are expected to rehabilitate the esthetic need of the patient and concurrently should not ignore the correction of deficiencies at the palatal surface of maxillary anterior teeth. The accurate palatal surface morphology of the maxillary anterior teeth is critical for establishing the incisal guidance. Unattended compromised anterior guidance for longer durations can initiate attrition and abfraction of posterior teeth, periodontal diseases due to trauma from occlusion, and temporomandibular joint disorders. This case report describes the rehabilitation of intrinsic dental erosion with indirect all-ceramic restorations to restore the esthetics, anterior guidance, and stable occlusion.

## 2. Case Presentation

**Figure 1:**
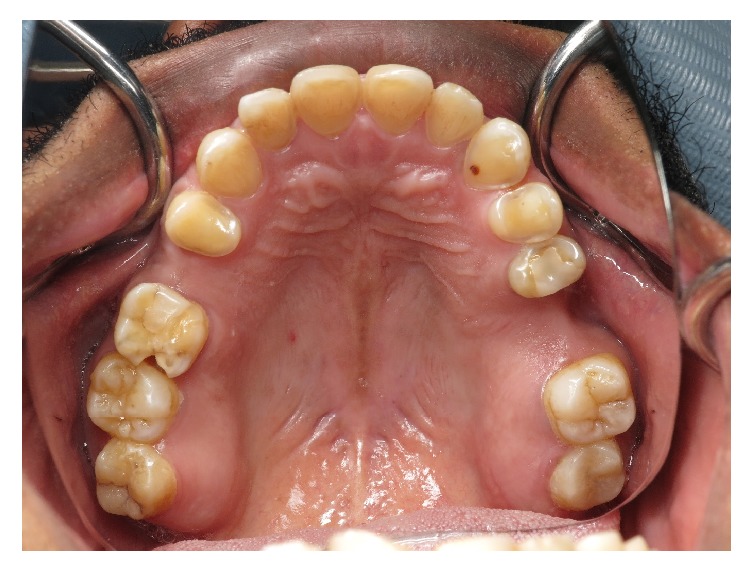
Dental erosions on the palatal surface of the maxillary anterior teeth and premolars.

**Figure 2:**
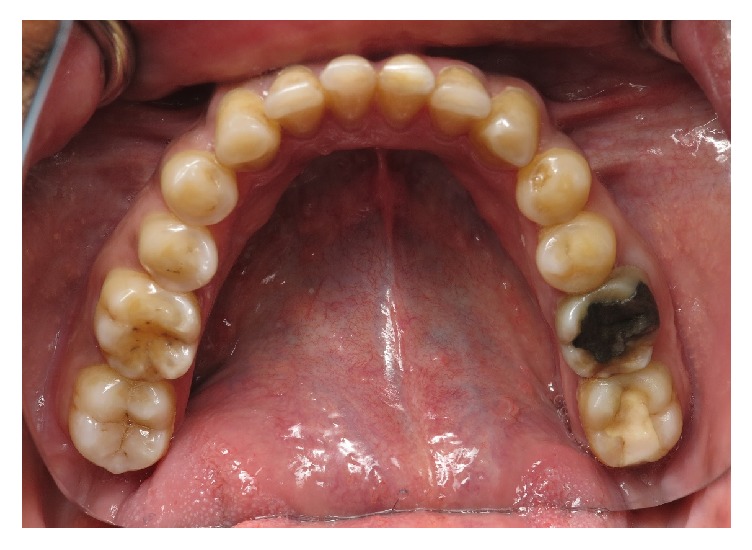
Dental erosions on cusps of canines and premolars in the mandibular arch.

**Figure 3:**
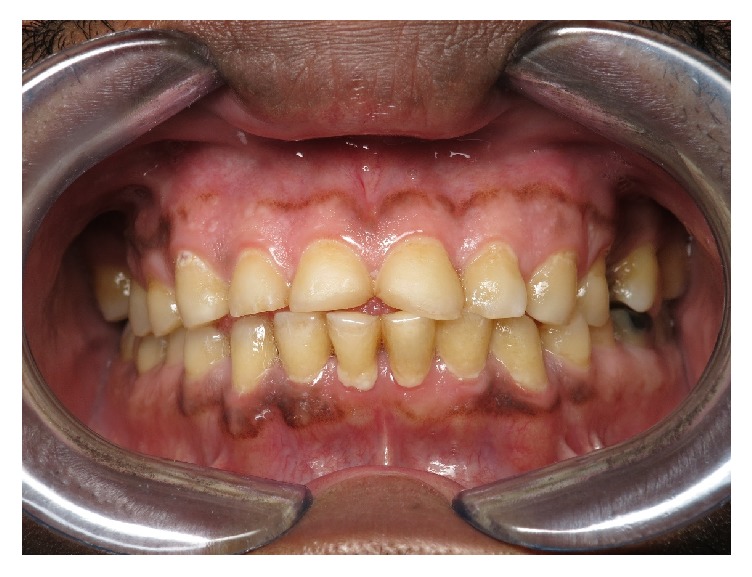
Short, unesthetic maxillary anterior teeth.

**Figure 4:**
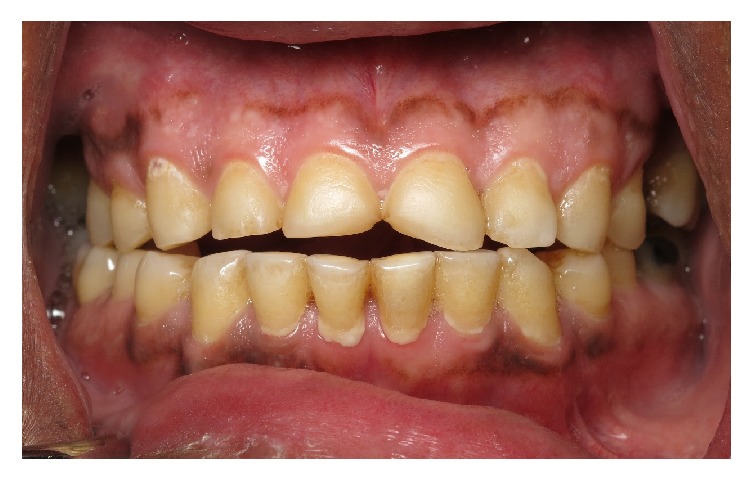
Protrusive interference and lack of anterior guidance.

A 54-year-old patient of Saudi Arabian nationality was referred to the postgraduate clinics, Faculty of Dentistry, King Khalid University, with a chief complaint of spontaneous pain in the upper left tooth along with compromised esthetics and functions of dentition. He worked as an accountant in the office, with noncontributory medical history. Extraoral examination revealed tenderness on palpation over the right lateral pterygoid muscle. The temporomandibular joint showed no clicking and deviation on mouth opening. Intraoral examination revealed poor oral hygiene and generalized marginal gingivitis. The patient had missing maxillary right first molar and maxillary left second premolar. The patient also had multiple restorations and root canal treated teeth in the mouth. The spontaneous pain was due to the deep distal caries with pulpal involvement of the left first maxillary molar. The palatal aspect of the maxillary anterior teeth showed extensive erosive damage with dentin core exposure (Figure 1). The erosion lesion was located above the enamel-cementum junction with intact enamel along the gingival margin. Flattened cusp morphology with glossy occlusal surfaces was observed in maxillary premolar and first molar teeth. The teeth in the mandibular arch also showed initial dental erosion on canine and premolar teeth (Figure 2). The maxillary anterior teeth showed a thin incisal edge, incisal wear, and reduced clinical crown length. Esthetics were also compromised due to inappropriate width/length proportion at 117–105% (Figure 3). Occlusal analysis showed the balancing side interferences on the maxillary right second molar and left first molar. The lack of incisal guidance induced protrusive interference on bilateral posterior teeth (Figure 4). Free-way space evaluation confirmed the preservation of vertical height of occlusion.

Detailed enquiry on oral and parafunctional habits to assess the possible exposure to dental erosion revealed that the patient was smoking around 20 cigarettes per day, with a history of chronic gastric regurgitation. The patient was self-medicating with histamine-2 blockers (ranitidine) at the time of severe bouts and consuming 3-4 cans of soft drinks to overcome the smoking induced mouth dryness.

The patient was advised to consult his physician for further diagnosis and treatment of chronic gastritis. Gastroesophageal reflux was verified with the diagnostic report from the gastroenterologist. Barium esophagram and esophagogastroduodenoscopy diagnostic tests were conducted by the gastroenterologist for the confirmation of GERD. The identification of etiologic agents and their elimination are critical to stop further damage to the tooth structure.

The full-arch impression for both jaws was made from irreversible hydrocolloid material (Cavex Impressional, Cavex Holland). Diagnostic casts were mounted on the semiadjustable articulator (Hanau 3040, Whip Mix Corp., Louisville, USA) with the help of interocclusal and face bow (Hanau Earpiece Facebow, Whip Mix Corp., Louisville, USA) records. The centric and dynamic occlusal contacts were tested with diagnostic mounting on the articulator. After thorough clinical, radiographic, and diagnostic cast evaluation, the patient was diagnosed with an erosive tooth surface loss due to GERD. The patient was also suffering from myofascial pain, lack of anterior guidance, and functional occlusal interferences. The main objective of the treatment was comprehensive esthetics and functional rehabilitation by restoring the maxillary anterior teeth, replacing the missing teeth, and reestablishing stable occlusion. Various treatment modalities were discussed in detail, and informed consent was obtained for the treatment plan. Due to the socioeconomic constraints, the patient opted to replace the missing teeth with a conventional tooth supported fixed partial denture instead of implant supported prosthesis.

**Figure 5:**
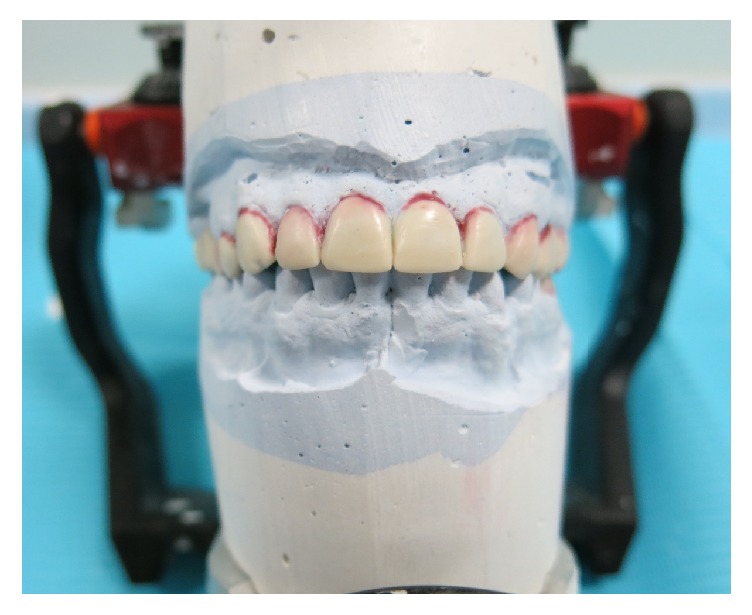
Diagnostic wax-up over the semiadjustable articulator.

**Figure 6:**
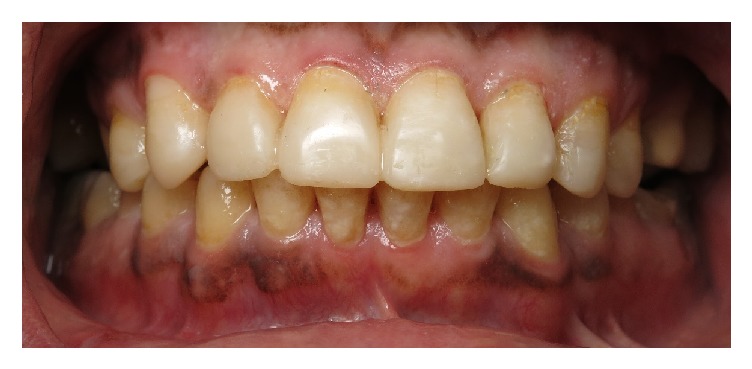
Composite build-up as long-term temporization.

**Figure 7:**
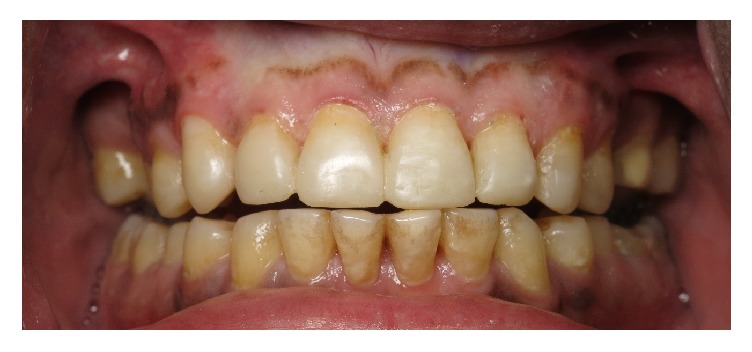
Improved incisal/anterior guidance with composite build-up.

**Figure 8:**
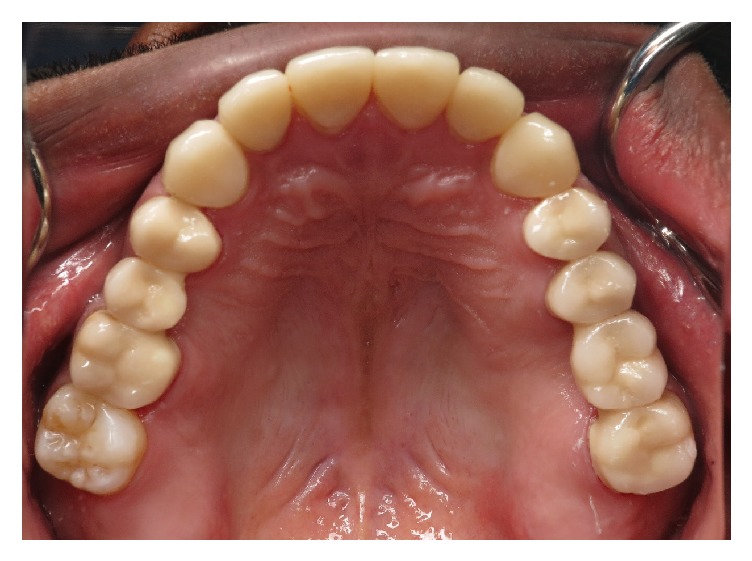
Zirconia crown and bridges on restoring maxillary arch.

**Figure 9:**
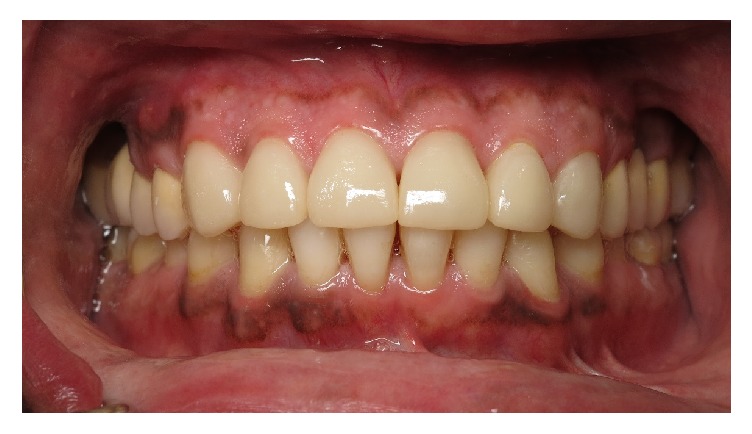
Final prosthesis with improved esthetics and anterior guidance.

The diagnostic wax-up (Figure 5) was done to accomplish multiple objectives like establishing an accurate incisal guidance and verifying the esthetic and functional modification of dentition. The occlusal plane was established with the help of Broadrick occlusal plane analyzer (Whip Mix Corp., Louisville, USA). The condylar guidance was set with the help of centric and eccentric interocclusal records. The incisal-cervical length of the incisors was increased to improve the incisal guidance as well as to correct width/length proportion. The accuracy of anterior guidance was verified on the articulator by eliminating the posterior interferences. The diagnostic wax-up procedure was completed on posterior teeth from both arches. The crown on root canal treated left lower molars assisted in reestablishing the occlusal plane and in eliminating the interferences. The silicone putty indexes of the diagnostic wax-up were utilized to restore the maxillary anterior teeth with a direct composite material (Filtek Z250; 3M ESPE, St. Paul, USA) (Figure 6). The incisal overlap and incisal edge position were further refined with visibility and phonetic methods (Figure 7). The composite restorations were polished and minor occlusal corrections were made on maxillary molars. The patient was recalled every week for 30 days to evaluate the stability of occlusion, adaptability of TMJ, and muscle of mastication for the modified occlusion. During one-month follow-up, the tenderness on the left lateral pterygoid muscle was eliminated with no TMJ discomfort. The alginate impressions were made again and mounted on the semiadjustable articulator with the help of interocclusal and face bow records. The customized incisal table was fabricated on the articulator to preserve the incisal guidance records during construction of the final prosthesis. The maxillary anterior teeth from canine to canine was prepared for monolithic full contour zirconia crowns. The maxillary premolar and molars were prepared as abutments to replace the missing maxillary left first molar and right second premolar. The final impression was made with additional silicone putty wash impression method after gingival retraction. The provisional restorations (Luxatemp, Hamburg, Germany) were made by an indirect method in the laboratory and cemented with noneugenol zinc oxide temporary cement (RelyX Temp NE, 3M ESPE, St. Paul, USA). Working cast obtained from the final impression was remounted on the existing articulator with intact customized incisal table. The palatal surfaces of final restorations were replicated similar to the direct composite temporization with the aid of an existing customized incisal table. The incisal edge position and contours were simulated with the help of silicone putty indexes from the composite restorations (Figure 8). The final crowns and fixed partial dentures (Figures 8 and 9) were cemented in self-adhesive composite resin cement (RelyX Unicem, 3M ESPE, St. Paul, USA). Occlusal refinement was performed over two follow-up appointments with one-week interval. The patient was counseled about the oral hygiene and dietary and lifestyle amendments.

**Figure 10:**
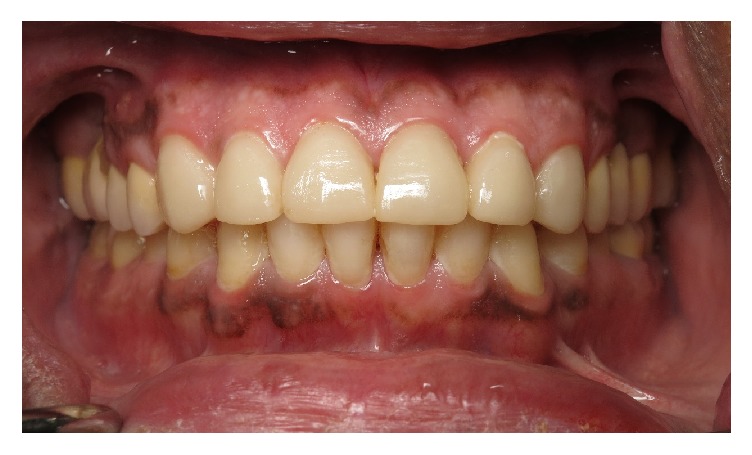
Six-month postoperative follow-up.

The patient was evaluated postoperatively after six months (Figure 10). Clinical examination revealed no abnormal clinical signs, and the patient showed good adaptation to new restorations. The pain from the left lateral pterygoid muscle was also relieved. The patient is now under the care of a gastroenterologist, and currently symptoms of GERD are relieved with medication and change in habits and lifestyle. The patient stopped the smoking habit and frequent consumption of soft drinks. During the follow-up appointments, patient self-report was sought to confirm the adherence to medication and amended lifestyle.

## 3. Discussion

Dental erosion is a common oral condition, and it is becoming endemic in recent decades. The majority of cases belong to four groups [19]: young women with eating disorders like bulimia and anorexia nervosa, teenage males with increased consumption of soft drinks, middle-aged men due to GERD, and the elderly population as a result of xerogenic medications. Early detection and intervention are strongly recommended to prevent extensive damage to the dentition. Reports suggest the high incidence of GERD in middle-aged men. It is attributed to work and personal stress, dietary habits, and family history. The dentist may be the first professional to suspect GERD due to dental erosion. The regurgitated gastric contents with pH below 1 have a severe demineralizing effect on the tooth structure. The patient seldom reports in early stages due to affected parts at the invisible areas like palatal surfaces of maxillary incisors and mandibular molars.

The frequent consumption of soft or sport drinks is known to escalate dental caries and enamel erosion activities. Soft drinks are reported to have lower pH and reduced buffering capacities, and carbonated drinks decrease the surface hardness of enamel, dentin, and composite restorations [20]. Both frequencies of intake and habit of holding the drink for a longer period in the mouth lead to pronounced dental erosion. Researchers report that frequent and long-term tobacco smoking significantly reduces the resting whole-mouth salivary flow rate [21]. Earlier investigations have pointed at the importance of normal salivary flow in preventing dental disorders. The buffering capacity of the saliva helps in the formation of protective enamel pellicle and prevents dental decalcification [22]. The location of the dental erosion will provide the cue to the dentist in estimating etiological factors. The extrinsic erosion is routinely observed on the labial surfaces of maxillary anterior teeth, while the intrinsic erosion lesions often present in the palatal surface of anterior teeth [23]. Careful extraoral examination is necessary for the dental erosion patient to evaluate the temporomandibular joints and muscles of mastication [24]. It includes the evaluation of the joint and muscle tenderness, clicking, deviation, deflection, and limitation of the mouth opening.

Treatment options for dental erosion differ according to the extent of tooth structure damage. Hence, selecting restorative options required the analysis of remaining tooth structure, location of tooth loss, and occlusion. The treatment choice ranges from a conventional fixed and removable prosthesis to more conservative, less invasive adhesive restorations. The patients affected by severe erosive destruction need complex occlusal rehabilitation. The placement of extensive restorations like porcelain veneers only and full veneer crowns is utilized. Interceptive treatments are required for correction of esthetic impairments, functional difficulties, and pain or sensitivity of teeth.

Dental erosion can affect the whole dentition but is most often limited to anterior teeth. The availability of space for restorations at the centric occlusion position and the presence of guiding contacts in eccentric mandibular movements should be evaluated during restoration of localized wear [25]. The resin-bonded palatal metal alloy veneers and direct composite restoration are recommended for palatal surface wear. The blanching of the incisal edge is the main drawback of palatal metal alloy veneer [26]. Direct acid-etch retained composite resins are advocated to restore the incisal-palatal surface wear. Alternatively, indirect composite resin and modified porcelain laminate veneer restorations [27, 28] are also employed due to improved physical properties and better procedural control. These procedures are clinically challenging due to the difficulty in hiding the junction between the natural teeth to restoration and consequently compromised esthetics. Dental wear involving the labial-incisal-palatal surfaces requires complex restorations like labial ceramic laminate veneer and lingual metal alloy veneer [29], resin-bonded minimum ceramic crown [30], or metal-ceramic crowns [31]. It is advisable to preserve the enamel during full crown preparation in teeth affected with dental erosion, since the enamel bonding to composite or porcelain restoration is predicable and long-lasting in comparison to dentin [32].

Treatment options depending on the loss of vertical dimension are described by Schuyler [33]. Direct composite restorations are recommended for vertical dimension loss of less than 2 mm, while indirect ceramic veneer and overlays are recommended for more than 2 mm loss in vertical dimension. Indirect ceramic restorations are suggested for the rehabilitation of erosion with loss of vertical dimension more than 4 mm. Conventional crowns are effective in restoring extensively worn teeth, replacing missing teeth, and overcoming reduced crown height. They offer additional advantages like providing provisional restorations to evaluate the esthetics and function for both the dentist and the patient.

The critical part of dental erosion management includes the prevention of further dental wear. Hence, the etiology of dental erosion should be determined to stop the disease and consequently the erosive process. Teamwork with medical professionals is mandatory along with restorative rehabilitation in treating structural damage from intrinsic erosions.

Zirconia is less reactive to the acidic environment compared to the direct composite restorations and more esthetic than metal-ceramic restorations. The extensive palatal surface erosions in maxillary anterior teeth reduce the incisal height and affect the horizontal overlap and the anterior guidance. Hence, it is strongly recommended to reestablish accurate incisal guidance during restorative procedures on anterior teeth [34]. Inadequate anterior guidance induces posterior interferences in mandibular eccentric movements. The disclusion of posterior teeth during mandibular eccentric movement reduces the posterior teeth contact time and precludes the consequent deleterious outcomes like attrition, fracture of posterior teeth, and periodontal diseases due to trauma from occlusion. Digital occlusal evaluation with T-Scan indicates the increased disclusion time [35] in patients with deficient incisal guidance. Researchers also reported lesser masticatory muscle activity with canine guided occlusion with adequate incisal guidance [36] and help in relieving myofascial pain. The increased disclusion time, with balancing interferences, is reported to initiate temporomandibular disorders [37].

The diagnostic mockup with composite temporization for a longer duration will provide the opportunity to elicit the opinion of the patient regarding the tooth form, shape, and inclination. It is a form of visual aid for the dentist to evaluate the esthetics and phonetics. Temporary restorations also help in assessing the response from the muscles of mastication and TMJ to the newly established occlusion [26].

Patients with dental erosion require counseling to change the dietary and lifestyle issues. Postoperative follow-up care is important to evaluate, monitor, and reemphasize the behavioral changes, along with required modifications of the prosthesis.

## 4. Conclusions

There are an ever-increasing number of patients with dental erosions in the society. Early diagnosis and preventive measures to avoid severe structural loss of the enamel and dentin are strongly recommended. Comprehensive diagnosis to identify the etiological factor and extent of tooth structure loss will enable the dentist to select the proper treatment plan and choose the appropriate material for long-term success.

Posttreatment follow-up and counseling are essential to motivate the patient to follow a healthy lifestyle for extended positive prognosis.
